# Rat Nasal Respiratory Mucosa-Derived Ectomesenchymal Stem Cells Differentiate into Schwann-Like Cells Promoting the Differentiation of PC12 Cells and Forming Myelin *In Vitro*

**DOI:** 10.1155/2015/328957

**Published:** 2015-08-03

**Authors:** Jian Zhang, Xin Gao, Hongjun Zou, Jinbo Liu, Zhijian Zhang

**Affiliations:** ^1^Department of Orthopedics, The Third Affiliated Hospital of Suzhou University, Changzhou 213003, China; ^2^School of Medical Science and Laboratory Medicine, Jiangsu University, Zhenjiang 212013, China

## Abstract

Schwann cell (SC) transplantation as a cell-based therapy can enhance peripheral and central nerve repair experimentally, but it is limited by the donor site morbidity for clinical application. We investigated weather respiratory mucosa stem cells (REMSCs), a kind of ectomesenchymal stem cells (EMSCs), isolated from rat nasal septum can differentiate into functional Schwann-like cells (SC-like cells). REMSCs proliferated quickly *in vitro* and expressed the neural crest markers (nestin, vimentin, SOX10, and CD44). Treated with a mixture of glial growth factors for 7 days, REMSCs differentiated into SC-like cells. The differentiated REMSCs (dREMSCs) exhibited a spindle-like morphology similar to SC cells. Immunocytochemical staining and Western blotting indicated that SC-like cells expressed the glial markers (GFAP, S100*β*, Galc, and P75) and CNPase. When cocultured with dREMSCs for 5 days, PC12 cells differentiated into mature neuron-like cells with long neurites. More importantly, dREMSCs could form myelin structures with the neurites of PC12 cells at 21 days *in vitro*. Our data indicated that REMSCs, a kind of EMSCs, could differentiate into SC-like cells and have the ability to promote the differentiation of PC12 cells and form myelin *in vitro*.

## 1. Introduction

Schwann cells (SCs) are myelin-forming glial cells in peripheral nervous system (PNS), and they have been reported to support nerve regeneration in both PNS and central nervous system (CNS) by forming myelin and providing various neurotrophic factors and molecular anchors [1–4]. In PNS, end-to-end anastomosis has been recommended as the primary choice for the treatment to acute transection injury of nerve tissue. When the gap between two damaged nerve ends is too large to be sutured, autologous nerve grafts are usually required [5]. Limited supply of autologous nerve tissue and the morbidity of the donor site are challenges to the neurologists. In CNS, spinal cord injury, representing common CNS damage and notorious for its life-long disability complication to the patients, is still lacking effective cure method both scientifically and therapeutically [6]. Tissue engineering techniques which provide seed cells for nerve injury could be an alternative repair strategy. Successful tissue engineered regeneration of nerve tissue have been reported using Schwan cells, a variety of cell carriers and cytokines and growth factors, which may open a way for therapeutic cure of damaged nerve tissue [7–16].

However, serious concerns for using SCs to tissue-engineering nerve tissue are the invasive approaches to collect SC-donor tissue and the difficulties to culture expand them [17]. Therefore, expansion and induction of stem cells* in vitro *are considered a promising tool to overcome the practical and ethical concerns of tissue transplantation, and actively searching for an appropriate source of cells has been the primary focus of nerve tissue engineering field.

Mesenchymal stem cells (MSCs) originating from mesoderm may be an alternative cell source for the generation of nerve tissue due to their multipotent differentiation properties. Some reports show that bone marrow-derived mesenchymal stem cells (BMSCs) and adipose-derived mesenchymal stem cells (ADSCs) can transdifferentiate into SCs [12, 18]. However, the neurogenic potential of MSCs is weaker when compared with those of stem cells derived from neural tissue, as they originate from the mesodermal layer [19, 20]. Although it has been shown that neural stem cells (NSCs) isolated from the brain of new born Sprague Dawley (SD) rats could differentiate into SC-like cells, human NSCs are difficult to be widely used in clinical practice because of the difficulties to obtain from allogeneic tissue sources and unavailability of autologous sources [21, 22].

Ectomesenchymal stem cells (EMSCs), which are pluripotent cells capable of self-renewal and differentiation into multiple cell types, are derived from the neural crest during embryonic development [23, 24]. During the embryonic development ectomesenchyme contributes to the formation of craniofacial structures. Nasal septum mucosa is composed of olfactory mucosa in the upper portion and respiratory mucosa in its lower part although they both arise from the embryonic ectoderm layer. In adult mammals, stem cells present in human nasal mucosa and can be induced to differentiate into neuron like cells [25]. Our previous work demonstrated that respiratory mucosa adjacent to the olfactory mucosa contains a population of EMSCs [26]. We also found that respiratory mucosa stem cells (REMSCs) were more amenable to differentiate into neural or glial cell compared to bone marrow-derived MSCs after a short period of neural induction culture [27]. Therefore, it is postulated that the REMSCs may have the potential to differentiate into SC-like cells.

In the present study, REMSCs were isolated, expanded, and identified as EMSCs and induced to differentiate into SC-like cells. The ability of promoting the differentiation of PC12 cells and forming myelin was also assessed* in vitro*.

## 2. Material and Methods

### 2.1. Cultivation of REMSCs and PC12 Cells

This study has been approved by the IACUC of Jiangsu University. The cultivation and isolation of REMSCs were performed as previously reported by Liu et al. [26] with minor modification. In brief, adult SD rats were anaesthetized with intraperitoneal injection of pentobarbital sodium (0.05 g/kg). Middle third of nasal septum was dissected, washed in DMEM/F-12 (DF12) (Gibco, USA) three times, and then cut into small pieces and digested with 0.25% trypsin (Gibco, USA) in phosphate buffered solution (PBS, Gibco, USA) for 25 min at 37°C. The tissue/cells suspension was placed into a 25 cm^2^ flask in growth medium that is DF12 supplemented with 10% FBS, 100 U/mL penicillin, and 100 *μ*g/mL streptomycin (Gibco, USA) and cultured at 37°C in 5% CO_2_ and 95% air with saturated humidity. The growth of the cells was recorded daily by digital camera connected to a phase-contrast microscope (Zeiss, Observer, A1). The medium was changed every 72 hours. When adherent cells had migrated from the explants and reached 80% confluent, generally one week in culture, cells were suspended with 0.05% trypsin-EDTA and reseeded in new culture flasks at 5 × 10^3^ cells/cm^2^ in the same growth medium for observation. Cells at their fourth passage were used for all of the characterization studies.

PC12 is a cell line derived from a pheochromocytoma of the rat adrenal medulla. This cell line is commonly used as a model system for neuronal differentiation in a culture set-up [28]. PC12 cells were cultured at 37°C and 5% CO2 in DF12 medium supplemented with 10% FBS, 100 U/mL penicillin, and 100 *μ*g/mL streptomycin (Gibco, USA).

### 2.2. Induction of Rat REMSCs into SC-Like Cells

REMSCs at their 4th passage were used to differentiate into SC-like cells. SC-like cells differentiation medium (SCDM) was DF12 medium supplemented with 10% FBS, 5 ng/mL platelet-derived growth factor-AA (PDGF-AA; PeproTech, USA), 10 ng/mL bFGF (PeproTech, USA), 5 *μ*M forskolin (Sigma, USA), and 200 ng/mL heregulin (HRG, PeproTech, USA). Cells were incubated for 10 days in SCDM with fresh medium added every 72 hours.

### 2.3. Coculture SC-Like Cells with PC12 Cells* In Vitro*

The ability of induced SC-like cells to promote the differentiation of PC12 cells was determined by examining their interaction with PC12 cells and the myelin-forming ability of SC-like cells. The PC12 cells were dissociated and replated at a density of 500 cells/cm^2^ in dishes in DF12 plus 10% FBS. After 24 hours, 4 groups of cocultures were established: group A: PC12 cells were cultured alone in DF12 plus 10% FBS; group B: PC12 cells were cultured in SCDM; group C: PC12 cells and 5000 cells/cm^2^ REMSCs were cocultured in DF12 plus 10% FBS; group D: PC12 cells and 5000 cells/cm^2^ SC-like cells were cocultured in SCDM. These 4 groups were cultured for 5 days and the mediums were replaced every 48 hours.

To observe the interaction of the two types of cell, SC-like cells were infected with green fluorescent protein (GFP) recombinant adenovirus, and PC12 cells were labeled by CM-Dil (Invitrogen, USA). GFP recombinant adenovirus was amplified in HEK 293 cell line. After REMSCs were cultured in differentiation medium for 1 week, GFP virus was added to infect the REMSCS at 100MOI for 24 hours. CM-Dil was added to PC12 cells culture medium at working concentration (1 *μ*M); PC12 cells were incubated in the culture medium with CM-Dil for 5 minutes at 37°C and then for an additional 15 minutes at 4°C. After the PC12 cells had been labelled, they were washed with PBS and resuspend in fresh medium for coculture with SC-like cells.

### 2.4. Western Blotting

Western blot analysis was used to detect the expression of P75, GFAP, CNPase, S100*β*, SOX10, nestin, vimentin, and CD44 by SC-like cells and REMSCs and the expression of NF-H, Synapsin II, GAP-43, and PSD-95 by PC12 cells. Total protein was extracted with RIPA buffer (10 mM Tris–HCl pH 7.8, 150 mM NaCl, 1% Nonidet P40, 0.1% sodium deoxycholate, 0.1% SDS, 1 mM EDTA, 8 M urea, 10 lg/mL aprotinin, and 1 mM PMSF). Protein concentration was determined by a bicinchoninic acid kit. Equal amounts of protein (50 *μ*g) from each sample were loaded onto 8% polyacrylamide gels, separated by 10% polyacrylamide gel electrophoresis, and electrophoretically transferred to PVDF membrane (Millipore, USA). Five% nonfat dry milk was used to block nonspecific binding of antibody. The membranes were incubated overnight at 4°C with either rabbit anti-P75 (1 : 500, Abcam, England), mouse anti-GFAP (1 : 400, Santa Cruz, USA), mouse anti-CNPase (1 : 500, Abcam, England), rabbit anti-S100*β* (1 : 1000, Abcam, England), rabbit anti-SOX10 (1 : 1500, Abcam, England), rabbit anti-vimentin antibody (1 : 1000, Abcam, England), rabbit anti-CD44 (1 : 500, Boster, China), anti-nestin antibody (1 : 500, Santa Cruz, USA), mouse anti-NF-H (1 : 300, Santa Cruz, USA), rabbit anti-Synapsin II (1 : 300, Santa Cruz, USA), mouse anti-GAP-43 (1 : 300, Santa Cruz, USA), rabbit anti-NGF (1 : 1000, Abcam, England), or rabbit anti-PSD-95 (1 : 500, Abcam, England) antibodies. The membranes were then incubated with HRP-conjugated goat anti-mouse or goat anti-rabbit secondary antibodies (1 : 1000, BioLegend, USA) for 1 hour. Membranes were treated with ECL chemiluminescent substrate (Millipore, USA) for 1 minute and developed by exposure to a cooled CCD camera (Sage Imaging System). Quantification of detected bands was performed by densitometry using ImageJ software.

### 2.5. Immunofluorescent Staining

Cells were fixed in 4% paraformaldehyde and then permeabilized with 0.1% Triton X-100/1% BSA in PBS. The primary rabbit anti-nestin antibody (1 : 300), rabbit anti-Vimentin antibody (1 : 200), rabbit anti-SOX10 (1 : 1000), rabbit anti-CD44 (1 : 200), anti-PSD-95 (1 : 1000), and anti-NF-H (1 : 300) were used to stain REMSCs for identification of EMSCs phenotype. The primary mouse anti-GFAP (1 : 300), rabbit anti-P75 (1 : 200), rabbit anti-S100*β* (1 : 300), rabbit anti-GALC (1 : 200, Santa Cruz, USA), and rabbit anti-CNPase (1 : 200) were used to stain SC-like cells for identification of SC phenotype. These cells were incubated at 4°C overnight with secondary antibodies including CY3-conjugated goat anti-mouse IgG (1 : 300, BioLegend, USA) and CY3-conjugated goat anti-rabbit IgG (1 : 300, BioLegend, USA) diluted in 1% BSA/PBS for 2-3 h at room temperature. Nuclei were labeled with Hoechst 33342 (Sigma, USA). The stained cells were examined with an inverted fluorescent microscope (Zeiss, Observer, A1, Germany).

### 2.6. Analysis of Neurite Outgrowth of PC12 Cells

After the PC12 cells were cocultured with SC-like cells infected with GFP or REMSCs infected with GFP for 5 days, morphological analysis and quantification of neurite bearing cells were performed under a fluorescent microscope as described previously [29, 30]. More than 100 cells in at ten randomly selected fields were counted and the cells with neurites greater than or equal to the length of its cell body were positive for neurite outgrowth. The positive cells were counted and expressed as a percentage of the total cells in each field. The neurite length was also measured for all the cells positive for neurite outgrowth in a field by tracing the longest length neurite. Average maximal neurite length per neurite-bearing cell in each field was calculated and data from the ten fields in each dish was designated as one experiment. The neurite length of neurite-bearing cells was measured by ImageJ software (NIH) [31] and recorded. These coculture experiments were repeated three times and analyzed independently.

### 2.7. Myelination Capacity of SC-Like Cells

PC12 cells were dissociated and replated at a density of 500 cells/cm^2^ in a culture dish and cultured in DF12 supplemented with 10% FBS. After 24 hours, SC-like cells were seeded at a density of 5000 cells/cm^2^ with PC12 cells and the medium was replaced with SCDM. As a control, the other two groups were designed: SC-like cells cultured alone, and REMSCs seeded with PC12 cells. The medium was changed every 72 hours. After 7 days in culture, the cells were fixed in 2% glutaraldehyde and then evaluated by scanning electron microscopy (Hitachi-S4800, Japan). After 21 days in culture, cells were fixed in 2% glutaraldehyde in sodium cacodylate buffer at 4°C for 24 hours, then fixed with 1% osmium tetroxide and 1% uranyl acetate, and embedded in epon. Ultrathin sections (50–70 nm) were cut and mounted on Formvar-coated slot grids. The ultrastructure of these cells was observed with transmission electron microscopy (Philips-Tecnai 12, Netherlands).

## 3. Statistical Analysis

Data were obtained from three separate experiments described above and present as mean ± SEM. One-way analysis of variance (ANOVA) with Dunnett's *T*3 test and Student's *t*-test was used to analyze the data. Values of *P* < 0.05 were considered to be statistically significant.

## 4. Results

### 4.1. Characteristics of REMSCs

**Figure 1:**
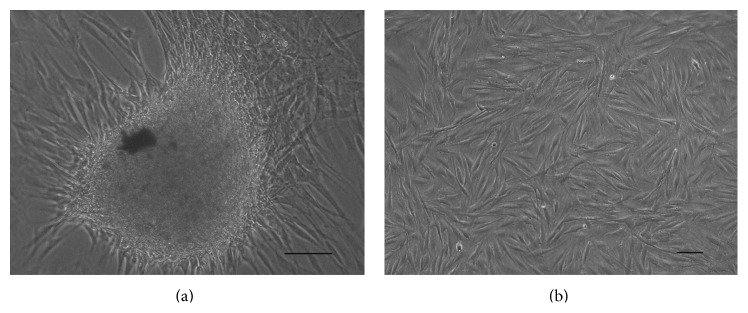
Cultivation and expansion: (a) primary cultured REMSCs at 5 days; cells migrated from the explants and attached to the culture plate. (b) Rat REMSCs at 4th passage demonstrated large and flat cell morphology. Bar: 50 *μ*m for all pictures.

**Figure 2:**
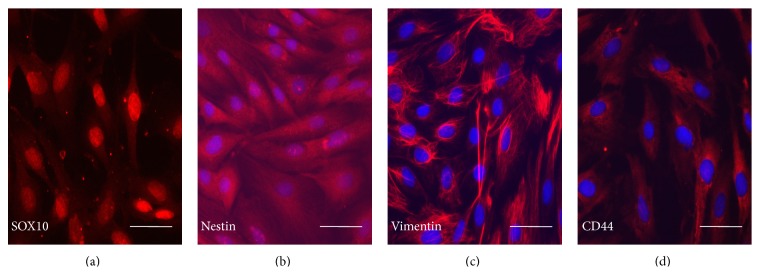
Labeling of neural crest cell markers on REMSCs: those markers were positively stained for SOX10 (a), nestin (b), vimentin (c), and CD44 (d). Nuclei were labeled with Hoechst 33342 (blue) except for SOX10. Bar: 20 *μ*m for all pictures.

After 5 days, adherent cells migrated from the explants and formed colonies (Figure 1(a)). REMSCs at 4th passage appeared as fibroblastic-like cells and proliferated rapidly on plastic plates (Figure 1(b)). Immunofluorescent staining showed almost all of the REMSCs expressed neural crest cell markers such as SOX10 (87.6 ± 0.7%), nestin (90.8 ± 0.8%), vimentin (92.2 ± 0.8%), and CD44 (88.1 ± 0.8%) (Figures 2(a)–2(d)).

### 4.2. Differentiation into SC-Like Cells

**Figure 3:**
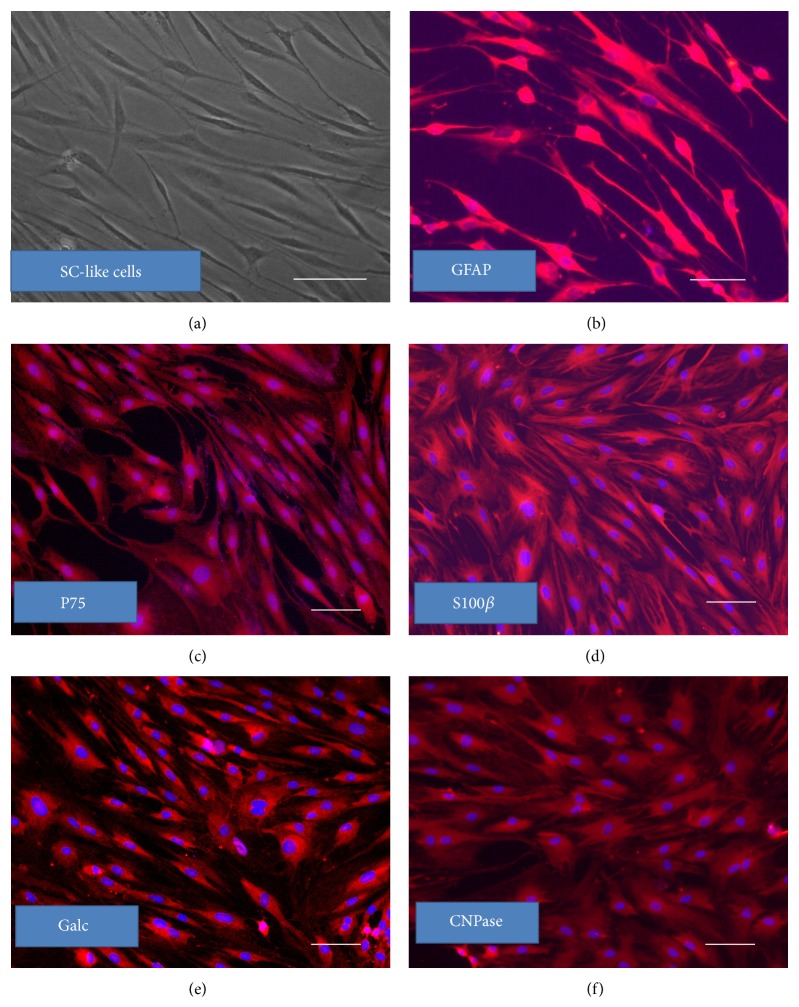
SC-like cells differentiated from REMSCs demonstrated typical Schwann cell morphology and were labeled with related cell surface markers. After 10 days, cells appeared typical bipolar and spindle-like Schwann cell phenotype (a), immunofluorescent staining showed that differentiated cells were stained positively with GFAP (b), P75 (c), S100*β* (d), Galc (e), and CNPase (f). Nuclei were labeled with Hoechst 33342 (blue). Bar: 50 *μ*m for all pictures.

REMSCs were treated with SCDM containing a mixture of glial cell growth factors for 10 days. The morphology of SC-like cells and the expression of the SC proteins such as GFAP, S100*β*, Galc, CNPase, and P75 were examined. After inductive differentiation, rat REMSCs changed from fibroblast-like morphology to spindle shape that seems to be more elongated than before, and these cells could continue to proliferate (Figure 3(a)). Immunofluorescent staining showed that about 79 ± 1.2% of the differentiated cells were positive for GFAP (Figure 3(b)); 81.7 ± 1.0% of the differentiated REMSCs were positive for P75 (Figure 3(c)); 89.2 ± 1.6% of the differentiated REMSCs were positive for S100*β* (Figure 3(d)); 84.9 ± 0.9% of the differentiated REMSCs were positive for Galc (Figure 3(e)), 84.6 ± 1.9% of the differentiated REMSCs were positive for CNPase (Figure 3(f)).

**Figure 4:**
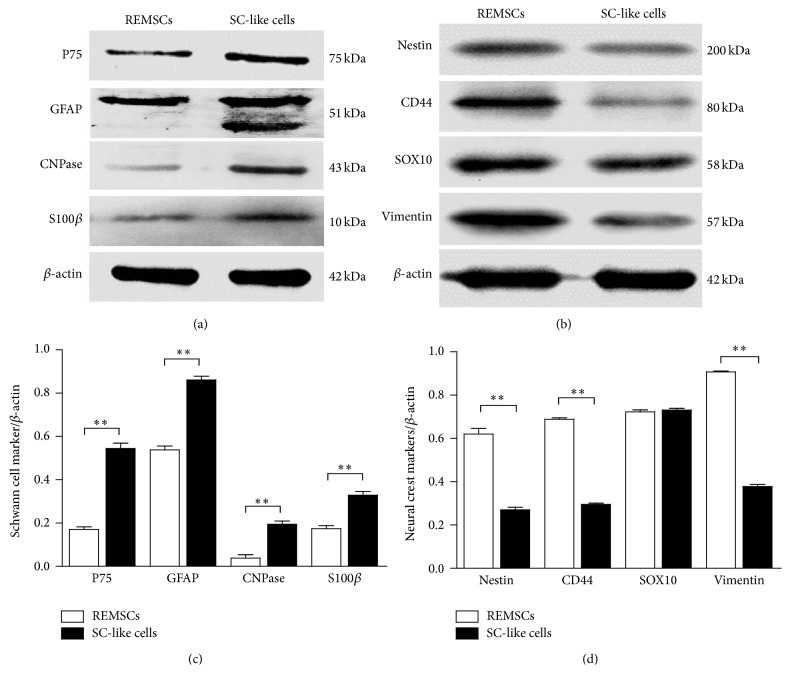
Western blotting showed the upregulation of expression of Schwann cell markers and the downregulation of expression of neural crest markers except for SOX10. Expressions of Schwann cell markers including GFAP, CNPase, P75, and S100*β* by REMSCs and SC-like cells were shown in (a); expressions of neural crest markers including SOX10, nestin, vimentin, and CD44 by REMSCs and SC-like cells were shown in (b); the experiments were replicated three times, and *β*-actin was used as a loading control. Quantitation of each marker was calculated using morphometric analysis with ImageJ software. Each bar showed the ratio of the expression level of marker protein to *β*-actin (c, d). The data were presented as mean ± SEM of three independent experiments. ^**^ *P* < 0.01 represented significant differences when compared between RMSCs and SC-like cells.

To further confirm the immunofluorescent staining results, Western blot analysis was used to examine the expression of glial specific markers and neural crest markers (Figure 4). *β*-Actin was used as a loading reference. The expression level of each protein was expressed as the ratio of the expression level of the marker protein to *β*-actin. The expression levels of GFAP, CNPase, P75, and S100*β* in SC-like cells were more pronounced compared to REMSCs (*P* < 0.01) (Figures 4(a) and 4(c)). In addition, Figures 4(b) and 4(d) showed the downregulation of nestin, vimentin, and CD44 proteins in SC-like cells (*P* < 0.01). However, the level of SOX10 was similar in REMSCs and SC-like cells (*P* > 0.05) (Figures 4(b) and 4(d)).

### 4.3. Functional Analysis of SC-Like Cells

**Figure 5:**
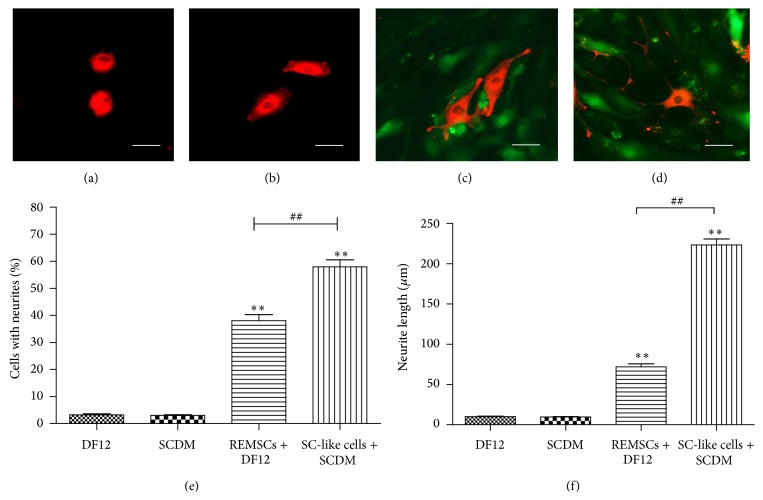
Neurite outgrowth by PC12 cells was observed in different conditions. PC12 cells were labelled by CM-Dil (red) and SC-like cells or REMSCs were infected with GFP virus (green). (a) PC12 cells were cultured alone in DF12 medium. (b) PC12 cells were cultured in SCDM alone. (c) PC12 cells were cocultured with REMSCs in DF12 medium. (d) PC12 cells were cocultured with SC-like cells in SCDM. PC12 cells with few neurites in (a) and (b) were round red. PC12 cells with neurites were observed in (c) and (d), and the cells in (d) showed morphological phenotype of mature neuron. The percentage of cells with neurites (e) and the length of neurites (f) were shown in the two bar graphs, respectively. Data were presented as mean ± SEM from three independent experiments. ^**^ *P* < 0.01 represent significant differences compared between group C or group D and group A; ^##^ *P* < 0.01 represent significant differences compared between groups C and D. Bar: 50 *μ*m for all figures.

**Figure 6:**
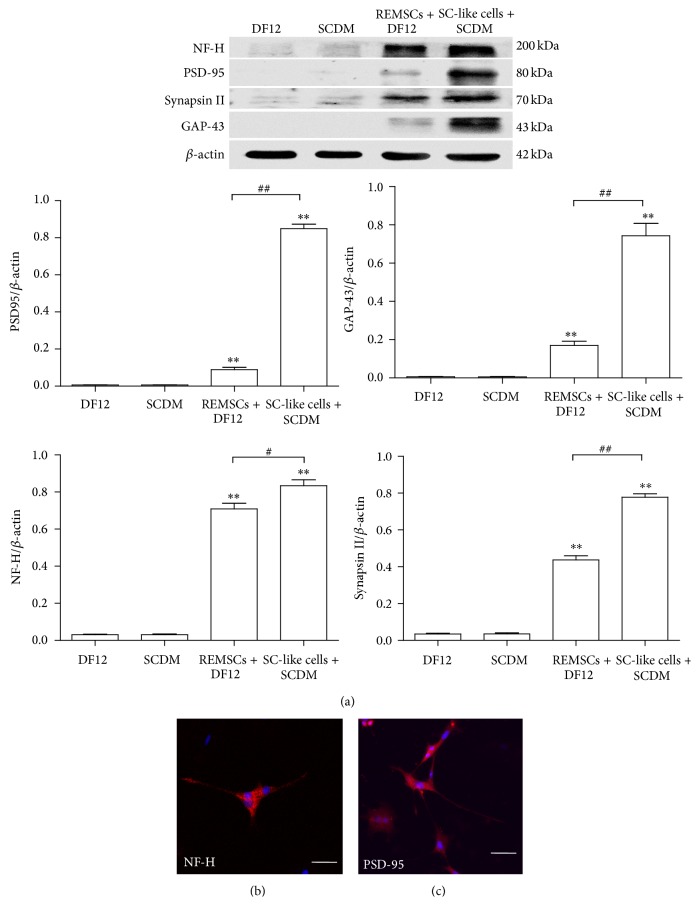
Western blot and immunofluorescent labeling indicated that SC-like cells promoted the differentiation of PC12 cells into mature neuron-like cells. (a) Neural cell markers including NF-H, GAP-43, PSD-95, and Synapsin II were detected in PC12 cells after being cultured for 5 days for all four groups, which were PC12 cells treated with DF12 medium, PC12 cells treated with SCDM, PC12 cells treated with DF12 medium and REMSCs, and PC12 cells treated with SC-like cells and SCDM. *β*-Actin was used as a loading control. The experiments were replicated three times and a representative blotting was shown. Each bar showed the ratio of marker protein to *β*-actin. The data were presented as the mean ± SEM of three independent experiments. ^**^ *P* < 0.01 represent significant differences compared with group A; ^##^ *P* < 0.01 represent significant differences compared with group C. Immunofluorescent staining showed that differentiated PC12 cells in group D expressed NF-H (b) and PSD-95 (c). Bar: 50 *μ*m for all pictures.

**Figure 7:**
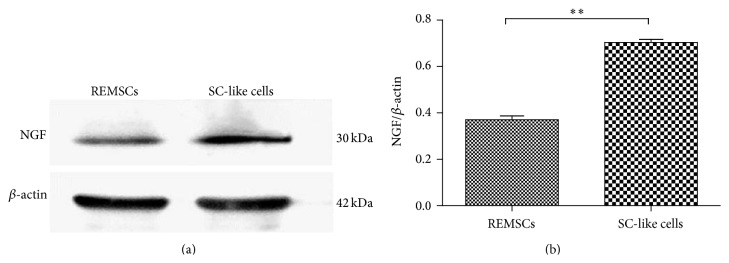
Expression levels of NGF by REMSCs and SC-like cells were observed and quantitated by Western blot. Significant differences between REMSCs and SC-like cells were observed (^**^ *P* < 0.01). Data were presented as mean ± SEM from three independent experiments.

**Figure 8:**
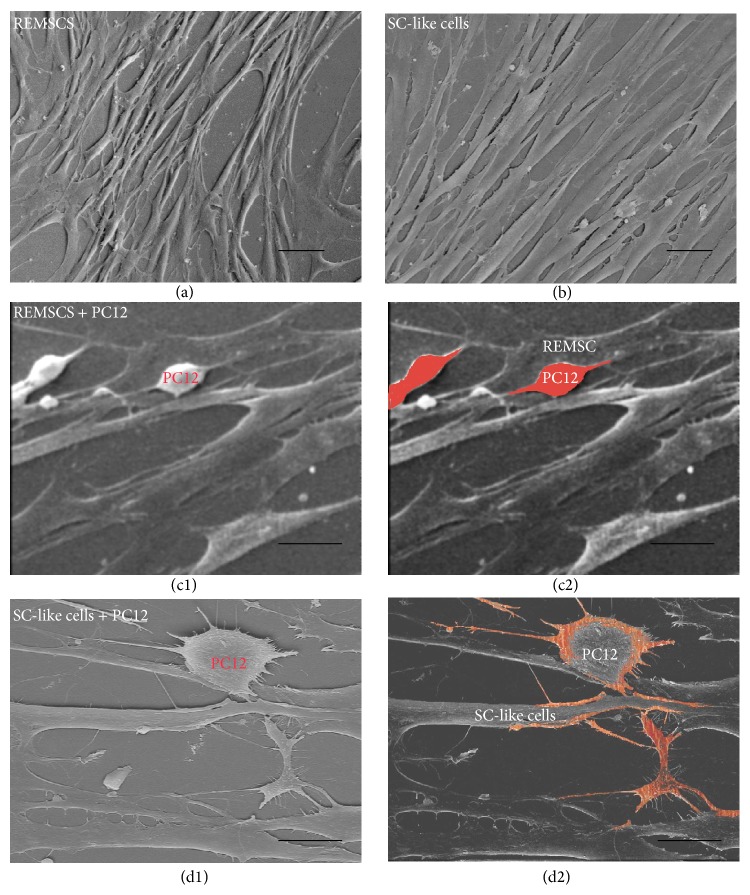
Morphology of REMSCs, SC-like cells, and PC12 cells cocultured with REMSCs or SC-like cells was observed under SEM. REMSCs cultured in DF12 medium (a) and SC-like cells cultured in SCDM (b), PC12 cells (marked with red) cocultured with REMSCs in DF12 medium (c1, c2), and PC12 cells (marked with red) cocultured with SC-like cells in SCDM (d1, d2). The imaging showed that the neurites of PC12 cells in (d1, d2) were longer than that in (c1, c2), and the neurites grew along with SC-like cells. Bar: 20 *μ*m for all pictures.

**Figure 9:**
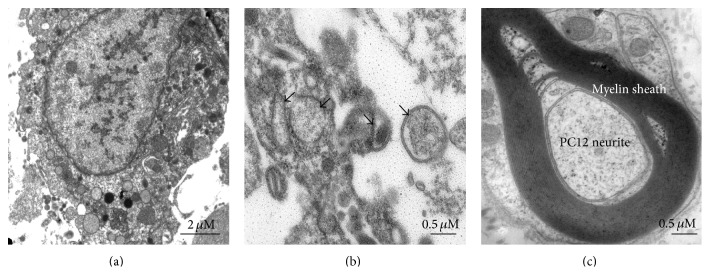
TEM observation showed that SC-like cells formed myelin sheath covering the neurites of PC12 cells (c). Myelin structures were not found when SC-like cells were cultured alone without PC12 cells (a). REMSCs could not form myelin structures with PC12 cells, and the arrows indicated that there was no myelin structure around the neurite. Bar: 2 *μ*m (a), 0.5 *μ*m ((b) and (c)).

To detect the ability of SC-like cells to induce the differentiation of PC12 cells and form myelin structures with the neurites of PC12 cells, we cocultured SC-like cells with PC12 cells which are neuron-like cells. PC12 cells were labeled with CM-Dil (Red); SC-like cells or REMSCs were infected with GFP virus (Green). PC12 cells in group A (PC12 cells cultured alone) and group B (PC12 cells cultured in SCDM) were round red and have few neurites (Figures 5(a) and 5(b)). In group C (PC12 cells and REMSCs were cocultured in DF12 medium), short neurites could be detected (Figure 5(c)), while, in group D (PC12 cells and SC-like cells were cocultured in SCDM), PC12 cells grew with long neurites (Figure 5(d)). Compared with group A (3.2 ± 0.4%), the percentage of positive neurite-bearing cells was significantly increased to 38.0 ± 2.3% (*P* < 0.01) and 57.9 ± 2.6% (*P* < 0.01), respectively, in group C and group D, but group B (3 ± 0.3%) (*P* > 0.05) had no significant difference (Figure 5(e)). Also, the percentage of positive neurite-bearing cells in group D significantly increased compared with group C (*P* < 0.01) (Figure 5(e)). Likewise, compared with group A (10.1 ± 0.5 *μ*m), the length of the longest neurite significantly increased to 72 ± 3.7 *μ*m (*P* < 0.01) and 223 ± 7.5 *μ*m (*P* < 0.01), respectively, in group C and group D (Figure 5(f)). There was no significant difference between group A and group B (9.7 ± 0.4 *μ*m) (Figure 5(f)). To further investigate the differentiation of PC12 cells, Western blotting was used to examine the expression levels of NF-H, Synapsin II, GAP-43, and PSD-95 in PC 12 cells. As shown in Figure 6(a), compared with group A, the level of GAP-43, NF-H, Synapsin II, and PSD-95 significantly (*P* < 0.01) increased in group C and group D. There was significant difference between group C and group D in NF-H, Synapsin II, PSD-95, and GAP-43 (*P* < 0.05). Immunofluorescent staining showed that differentiated PC12 cells in group D expressed NF-H (35.3 ± 0.42%) and PSD-95 (56.7 ± 0.47%) (Figures 6(b) and 6(c)). The level of NGF was examined as well. SC-like cells were strongly positive for NGF expression as compared with REMSCs (Figure 7). To assess the myelination capacity of SC-like cells, PC12 cells were cultured with SC-like cells or REMSCs. Consisting with the morphology observed under phase contrast microscopy, observation under scanning electron microscopy (SEM) also shows that SC-like cells were bipolar and spindle-like shaped (Figure 8(b)). When PC12 cells were cocultured with REMSCs the neurites were shorter (Figures 8(c1) and 8(c2)) and, in contrast, when cocultured with SC-like cells for 7 days, the neurites of PC12 cells were longer and grew along with SC-like cells (Figures 8(d1) and 8(d2)). After 21 days of coculture, transmission electron microscopy (TEM) showed that SC-like cells could form myelin sheath with neurites (Figure 9(c)). On the contrary, REMSCs could not form myelin structures with PC12 neurites (Figure 9(b)), and REMSCs could not form myelin structures without neurites (Figure 9(a)).

## 5. Discussion

Previous studies have shown that REMSCs could form neurospheres in neurosphere-forming condition and differentiate into neurons [32]. However, whether REMSCs could differentiate along a Schwann cell lineage is still unknown. Here, our results show that differentiated REMSCs have similar morphological and phenotypic characteristics as Schwann cells, and more importantly, differentiated REMSCs possess myelin-forming ability, which is the most important function of Schwann cells.

EMSCs originate from the neural crest during embryonic development and contribute to the formation of craniofacial structures [33]. In the head region, neural crest-derived stem cells can be found in a number of organs and tissues [34–40]. Recently, respiratory mucosa cells isolated from adult human inferior turbinate are reported to be multipotent neural crest-derived stem cells [41]. Our previous studies have shown that REMSCs expressed MSCs markers such as CD90, CD45, and CD105 can different into neuron-like cells and osteoblasts [25, 27]. A study from Goldstein and colleagues also shows that nasal stem cells derived from septum can form neurosphere and give rise to neuronal-like cells under differentiation conditions [32]. In the current study, immunofluorescent staining of REMSCs showed that most cells express neural crest markers including nestin, vimentin, and SOX10 (Figure 2). Nestin and vimentin are regarded as a marker of neural stem cells and expressed in neural crest cells [42–46]. SOX10 plays a role early in development when it is present in the neural crest cells, and it is the only transcription factor needed for the generation of glial cells from crest cells during the embryonic development [47]. Meanwhile, these cells also express CD44 that is the marker of premigratory and migratory cranial neural crest [48]. The coexpression of nestin, vimentin, SOX10, and CD44 provides strong evidence that REMSCs originate from neural crest and could be able to differentiate into glial cells and neuron-like cells.

It has been reported that stem cells from a variety of tissue sources were able to differentiate into SC-like cells [12, 18, 49]. Nestin-positive BMSCs have been observed to differentiate along the glial cell lineage [50, 51]. Minor percentile of adipose-derived stem cells was identified with positive nestin labeling and able to differentiate into SC-like cells [18, 52]. Also, it has been demonstrated that the glial formation potentials of MSCs derived from bone marrow and adipose may be explained by the presence of crest-derived cell subpopulation [53]. In addition, Labat et al. also found BMSCs originating from neural crest in the peripheral blood mononuclear cells, which simultaneously expressed the mesoderm markers and the neural ectoderm markers [54]. It suggests that ectomesenchymal-derived stem cells may exist in multiple tissues. However, the amount of the crest-derived cells in those tissues is too small to obtain sufficient SCs for effectively clinical application. The current study shows that almost all the REMSCs derived from nasal septum were nestin-positive and SOX10-positive. In agreement with our findings, REMSCs derived from human inferior turbinate also expressed neural crest markers [41]. Those results together suggest that REMSCs may have stronger potential to differentiate into SC-like cells than other source-derived stem cells.

To investigate the ability of REMSCs to differentiate into SC-like cells, REMSCs were cultured in a differentiation medium (HRG, FSK, PDGF-AA, and bFGF) which is previously used to induce Schwann cells from MSC and ADSC [18, 49, 55]. After 5 days, the REMSCs demonstrated elongated-spindle morphology. Western blot showed the downregulation of nestin, vimentin, and CD44 (Figures 4(b) and 4(d)). However, the level of SOX10 of SC-like cells is similar with REMSCs. SOX10 has been reported to be a neural crest marker [42]. Furthermore, SOX10 is important and expressed at all stages of Schwann cell development and works both independently and synergistically with other transcription factors to regulate Schwann cells specific loci [56–59]. In our study, western blot analysis showed that the level of SOX10 was similar in both REMSCs and SC-like cells. We speculate that SOX10 may play a role in the differentiation of REMSCs. Immunofluorescent staining of dREMSCs showed that most cells expressed Schwann cell markers such as GFAP, P75, and S100*β* (Figure 3). Similar results were also reported in previous studies [12, 18, 49]. Interestingly, SC-like cells expressed Galc which is a specific cell-surface antigenic marker for oligodendrocytes in culture [60]. Furthermore, these SC-like cells expressed CNPase (Figure 3), which was expressed in oligodendrocytes and Schwann cells. CNPase is regarded as marker for myelin-forming cells and photoreceptors for some neurons in long-term culture [61]. CNPase is both membrane bound and linked to microtubules, and it is the third most abundant myelin protein in the CNS, representing 4% of CNS myelin proteins [62]. Overexpression mutations show that CNPase plays a role in myelin compaction [63, 64]. Therefore, CNPase is considered to be a marker for the cells to produce myelin. These results suggested that the SC-like cells have the capability of myelination.

Evidence of morphological and phenotypic characteristics may not be enough to justify that the function of SC-like cells is similar to Schwann cells. It has been demonstrated that SC can induce the differentiation of PC12 cells and form myelin sheath with PC12 neurites [12]. Here, we tested the function of SC-like cells by being cocultured with PC12 cells. When cocultured with SC-like cells for 5 days, PC12 cells extended neurites and the percentage of cells with neurites significantly increased compared with that in coculture with REMSCs (Figure 5). Western blotting showed that SC-like cells promoted the expression level of NF-H, GAP-43, PSD-95, and Synapsin II in PC12 cells (Figure 6(a)). Immunofluorescent staining showed that differentiated PC12 cells expressed NF-H and PSD-95 (Figures 6(b) and 6(c)). NF-H provides stability to developing axonal neurites, and it is related to the stages of axonal outgrowth [65]. Also PSD-95, which is a membrane-associated guanylate, is the main scaffolding protein in the excitatory postsynaptic density [66]. All these results suggested that SC-like cells could promote the differentiation of PC12 cells. NGF is considered to play an important role in the differentiation of PC12. When PC12 cells are treated with NGF, PC12 cells extend neurites and form synapse structure and neurite network, differentiating into neuron-like cells [67]. In the current study, Western blotting showed that the expression level of NGF of SC-like cells was higher than that of REMSCs (Figure 7), which indicates that SC-like cells may promote the differentiation of PC12 cells by expression of NGF.

Myelin-forming ability is very important to SC cells. It is reported previously that SC-like cells induced from BMSCs and ADSCs can form myelin sheath* in vitro* [12, 52]. SC-like cells from REMSCs may have the ability to form myelin* in vitro* and* in vivo*. According to the “carpet crawler” model, myelination starts by the spreading of a membrane sheet along the neurite before it makes a turn and moves underneath the growing sheet [68]. We observed that SC-like cells grew along with the neurites of PC12 cells at 7 days (Figure 8) and formed myelin structures with neurites at 21 days (Figure 9(c)). However, myelin structures were not detected in SC-like cells cultured without PC12 cells and REMSCs cultured with PC12 cells. A previous study has demonstrates that Schwann cells become myelinating or nonmyelinating depending on the signal from axon [47]. Whether SC-like cells from REMSCs could form myelin* in vivo* is still a question to be answered in future studies though.

MSCs have been demonstrated to be safe, as they do not form tumor after transplantation [69]. Studies have shown that mouse embryonic stem cells (MESCs) would form teratocarcinomas when injected into immunodeficient mice [70]. Also, when injected into human embryonic stem cells (hESCs) in severe combined immunodeficient mice, hESCs could generate primitive, undifferentiated tumors [71]. On the contrary, Sieber-Blum found that epidermal neural crest stem cell grafted into the adult spinal cord does not form tumors [72]. Similarly, Stefan Hauser showed that neural crest stem cell from adult human inferior turbinate was not able to create teratoma [41]. Those data may collectively indicate the safety of using REMSCs and may suggest that transplantation of REMSCs could be an alternative cell-based therapeutic strategy for neurotissue engineering and neurodegenerative diseases. However, large scale preclinical and clinical studies on its safety are needed before any clinical application.

## 6. Conclusion

REMSCs isolated from nasal septum are able to differentiate into SC-like cells which have similar morphological, phenotypic characteristics, and function with Schwann cells. These findings may suggest that transplantation of REMSCs could be an alternative cell-based therapeutic strategy for neurotissue engineering and neurodegenerative diseases.

## Abbreviations

SC: Schwann cell
EMSCs: Ectomesenchymal stem cells
MSCs: Mesenchymal stem cells
BMSCs: Bone marrow-derived stem cells
ADSCs: Adipose-derived stem cells
REMSCs: Respiratory mucosa stem cells
SC-like cells: Schwann-like cells
DF12: DMEM/F-12
bFGF: Basic fibroblast growth factor
PDGF-AA: Platelet-derived growth factor-AA
dREMSCs: Differentiated REMSCs
GFAP: Glial fibrillary acidic protein
Galc: Galactosylceramidase
FBS: Fetal bovine serum
NGF: Nerve growth factor
GFP: Green fluorescent protein
SCDM: Schwann-like cells differentiation medium
MESCs: Embryonic stem cells
hESCs: Human embryonic stem cells.

## References

[1] Aguayo A. J., Attiwell M., Trecarten J., Perkins S., Bray G. M. (1977). Abnormal myelination in transplanted Trembler mouse Schwann cells. *Nature*.

[2] Aguayo A. J., Dickson R., Trecarten J., Attiwell M., Bray G. M., Richardson P. (1978). Ensheathment and myelination of regenerating PNS fibres by transplanted optic nerve glia. *Neuroscience Letters*.

[3] Bunge M. B. (1994). Transplantation of purified populations of Schwann cells into lesioned adult rat spinal cord. *Journal of Neurology*.

[4] Guest J. D., Rao A., Olson L., Bunge M. B., Bunge R. P. (1997). The ability of human schwann cell grafts to promote regeneration in the transected nude rat spinal cord. *Experimental Neurology*.

[5] Lundborg G. (2000). A 25-year perspective of peripheral nerve surgery: evolving neuroscientific concepts and clinical significance. *The Journal of Hand Surgery*.

[6] Tator C. H. (2006). Review of treatment trials in humanspinal cord injury: issues, difficulties, and recommendations. *Neurosurgery*.

[7] Bunge M. B., Wood P. M. (2012). Realizing the maximum potential of Schwann cells to promote recovery from spinal cord injury. *Handbook of Clinical Neurology*.

[8] Deng L.-X., Hu J., Liu N. (2011). GDNF modifies reactive astrogliosis allowing robust axonal regeneration through Schwann cell-seeded guidance channels after spinal cord injury. *Experimental Neurology*.

[9] Flora G., Joseph G., Patel S. (2013). Combining neurotrophin-transduced Schwann cells and rolipram to promote functional recovery from subacute spinal cord injury. *Cell Transplantation*.

[10] Ghosh M., Tuesta L. M., Puentes R. (2012). Extensive cell migration, axon regeneration, and improved function with polysialic acid-modified Schwann cells after spinal cord injury. *Glia*.

[11] Hadlock T., Sundback C., Hunter D., Cheney M., Vacanti J. P. (2000). A polymer foam conduit seeded with Schwann cells promotes guided peripheral nerve regeneration. *Tissue Engineering*.

[12] Keilhoff G., Stang F., Goihl A., Wolf G., Fansa H. (2006). Transdifferentiated mesenchymal stem cells as alternative therapy in supporting nerve regeneration and myelination. *Cellular and Molecular Neurobiology*.

[13] Wiliams R. R., Bunge M. B. (2012). Schwann cell transplantation: a repair strategy for spinal cord injury?. *Progress in Brain Research*.

[14] Xia L., Wan H., Hao S.-Y. (2013). Co-transplantation of neural stem cells and Schwann cells within poly (L-lactic-co-glycolic acid) scaffolds facilitates axonal regeneration in hemisected rat spinal cord. *Chinese Medical Journal*.

[15] Xu X., Geremia N., Bao F., Pniak A., Rossoni M., Brown A. (2011). Schwann cell coculture improves the therapeutic effect of bone marrow stromal cells on recovery in spinal cord-injured mice. *Cell Transplantation*.

[16] Zhou X.-H., Ning G.-Z., Feng S.-Q. (2012). Transplantation of autologous activated Schwann cells in the treatment of spinal cord injury: six cases, more than five years of follow-up. *Cell Transplantation*.

[17] Tohill M., Mantovani C., Wiberg M., Terenghi G. (2004). Rat bone marrow mesenchymal stem cells express glial markers and stimulate nerve regeneration. *Neuroscience Letters*.

[18] Kingham P. J., Kalbermatten D. F., Mahay D., Armstrong S. J., Wiberg M., Terenghi G. (2007). Adipose-derived stem cells differentiate into a Schwann cell phenotype and promote neurite outgrowth in vitro. *Experimental Neurology*.

[19] Raedt R., Pinxteren J., van Dycke A. (2007). Differentiation assays of bone marrow-derived Multipotent Adult Progenitor Cell (MAPC)-like cells towards neural cells cannot depend on morphology and a limited set of neural markers. *Experimental Neurology*.

[20] Song S., Song S., Zhang H., Cuevas J., Sanchez-Ramos J. (2007). Comparison of neuron-like cells derived from bone marrow stem cells to those differentiated from adult brain neural stem cells. *Stem Cells and Development*.

[21] Mothe A. J., Tator C. H. (2012). Advances in stem cell therapy for spinal cord injury. *The Journal of Clinical Investigation*.

[22] Tong L., Ji L., Wang Z., Tong X., Zhang L., Sun X. (2010). Differentiation of neural stem cells into Schwann-like cells in vitro. *Biochemical and Biophysical Research Communications*.

[23] Ishii M., Arias A. C., Liu L., Chen Y.-B., Bronner M. E., Maxson R. E. (2012). A stable cranial neural crest cell line from mouse. *Stem Cells and Development*.

[24] Janebodin K., Horst O. V., Ieronimakis N. (2011). Isolation and characterization of neural crest-derived stem cells from dental pulp of neonatal mice. *PLoS ONE*.

[25] Huang Q., Lu H., Zhou Y. (2012). Culture and induced multilineage differentiation of mesenchymal stem cells derived from human nasal mucosa. *Journal of Clinical Otorhinolaryngology, Head, and Neck Surgery*.

[26] Liu J., Chen Q., Zhang Z. (2013). Fibrin scaffolds containing ectomesenchymal stem cells enhance behavioral and histological improvement in a rat model of spinal cord injury. *Cells Tissues Organs*.

[27] Gao X., Zhang J., Zhang J., Zou H., Liu J. (2014). Identification of rat respiratory Mucosa stem cells and comparison of the early neural differentiation potential with the bone marrow mesenchymal stem cells in vitro. *Cellular and Molecular Neurobiology*.

[28] Greene L. A., Aletta J. M., Rukenstein A., Green S. H. (1987). PC12 pheochromocytoma cells: culture, nerve growth factor treatment, and experimental exploitation. *Methods in Enzymology*.

[29] Katoh S., Mitsui Y., Kitani K., Suzuki T. (1997). Hyperoxia induces the differentiated neuronal phenotype of PC12 cells by producing reactive oxygen species. *Biochemical and Biophysical Research Communications*.

[30] Lin C.-W., Wu M.-J., Liu I. Y.-C., Su J.-D., Yen J.-H. (2010). Neurotrophic and cytoprotective action of luteolin in PC12 cells through ERK-dependent induction of Nrf2-Driven HO-1 expression. *Journal of Agricultural and Food Chemistry*.

[31] Schneider C. A., Rasband W. S., Eliceiri K. W. (2012). NIH Image to ImageJ: 25 years of image analysis. *Nature Methods*.

[32] Goldstein B. J., Hare J. M., Lieberman S., Casiano R. (2013). Adult human nasal mesenchymal stem cells have an unexpected broad anatomic distribution. *International Forum of Allergy & Rhinology*.

[33] Santagati F., Rijli F. M. (2003). Cranial neural crest and the building of the vertebrate head. *Nature Reviews Neuroscience*.

[34] Brandl C., Florian C., Driemel O., Weber B. H. F., Morsczeck C. (2009). Identification of neural crest-derived stem cell-like cells from the corneal limbus of juvenile mice. *Experimental Eye Research*.

[35] Hunt D. P. J., Morris P. N., Sterling J. (2008). A highly enriched niche of precursor cells with neuronal and glial potential within the hair follicle dermal papilla of adult skin. *Stem Cells*.

[36] Techawattanawisal W., Nakahama K., Komaki M., Abe M., Takagi Y., Morita I. (2007). Isolation of multipotent stem cells from adult rat periodontal ligament by neurosphere-forming culture system. *Biochemical and Biophysical Research Communications*.

[37] Toma J. G., Akhavan M., Fernandes K. J. L. (2001). Isolation of multipotent adult stem cells from the dermis of mammalian skin. *Nature Cell Biology*.

[38] Waddington R. J., Youde S. J., Lee C. P., Sloan A. J. (2009). Isolation of distinct progenitor stem cell populations from dental pulp. *Cells Tissues Organs*.

[39] Widera D., Grimm W.-D., Moebius J. M. (2007). Highly efficient neural differentiation of human somatic stem cells, isolated by minimally invasive periodontal surgery. *Stem Cells and Development*.

[40] Widera D., Zander C., Heidbreder M. (2009). Adult palatum as a novel source of neural crest-related stem cells. *Stem Cells*.

[41] Hauser S., Widera D., Qunneis F. (2012). Isolation of novel multipotent neural crest-derived stem cells from adult human inferior turbinate. *Stem Cells and Development*.

[42] Chung K.-F., Sicard F., Vukicevic V. (2009). Isolation of neural crest derived chromaffin progenitors from adult adrenal medulla. *Stem Cells*.

[43] Dent J. A., Polson A. G., Klymkowsky M. W. (1989). A whole-mount immunocytochemical analysis of the expression of the intermediate filament protein vimentin in *Xenopus*. *Development*.

[44] Gilyarov A. V. (2008). Nestin in central nervous system cells. *Neuroscience and Behavioral Physiology*.

[45] Lothian C., Lendahl U. (1997). An evolutionarily conserved region in the second intron of the human nestin gene directs gene expression to CNS progenitor cells and to early neural crest cells. *The European Journal of Neuroscience*.

[46] Walker A. S., Goings G. E., Kim Y., Miller R. J., Chenn A., Szele F. G. (2010). Nestin reporter transgene labels multiple central nervous system precursor cells. *Neural Plasticity*.

[47] Bhatheja K., Field J. (2006). Schwann cells: origins and role in axonal maintenance and regeneration. *The International Journal of Biochemistry & Cell Biology*.

[48] Perissinotto D., Iacopetti P., Bellina I. (2000). Avian neural crest cell migration is diversely regulated by the two major hyaluronan-binding proteoglycans PG-M/versican and aggrecan. *Development*.

[49] Peng J., Wang Y., Zhang L. (2011). Human umbilical cord Wharton's jelly-derived mesenchymal stem cells differentiate into a Schwann-cell phenotype and promote neurite outgrowth in vitro. *Brain Research Bulletin*.

[50] Caddick J., Kingham P. J., Gardiner N. J., Wiberg M., Terenghi G. (2006). Phenotypic and functional characteristics of mesenchymal stem cells differentiated along a Schwann cell lineage. *Glia*.

[51] Wislet-Gendebien S., Bruyère F., Hans G., Leprince P., Moonen G., Rogister B. (2004). Nestin-positive mesenchymal stem cells favour the astroglial lineage in neural progenitors and stem cells by releasing active BMP4. *BMC Neuroscience*.

[52] Xu Y., Liu L., Li Y. (2008). Myelin-forming ability of Schwann cell-like cells induced from rat adipose-derived stem cells in vitro. *Brain Research*.

[53] Morikawa S., Mabuchi Y., Niibe K. (2009). Development of mesenchymal stem cells partially originate from the neural crest. *Biochemical and Biophysical Research Communications*.

[54] Labat M. L., Milhaud G., Pouchelet M., Boireau P. (2000). On the track of a human circulating mesenchymal stem cell of neural crest origin. *Biomedicine & Pharmacotherapy*.

[55] Kashani I. R., Golipoor Z., Akbari M. (2011). Schwann-like cell differentiation from rat bone marrow stem cells. *Archives of Medical Science*.

[56] Britsch S., Goerich D. E., Riethmacher D. (2001). The transcription factor Sox10 is a key regulator of peripheral glial development. *Genes & Development*.

[57] Kuhlbrodt K., Herbarth B., Sock E., Hermans-Borgmeyer I., Wegner M. (1998). Sox10, a novel transcriptional modulator in glial cells. *The Journal of Neuroscience*.

[58] Stolt C. C., Wegner M. (2010). SoxE function in vertebrate nervous system development. *The International Journal of Biochemistry & Cell Biology*.

[59] Svaren J., Meijer D. (2008). The molecular machinery of myelin gene transcription in Schwann cells. *Glia*.

[60] Raff M. C., Mirsky R., Fields K. L. (1978). Galactocerebroside is a specific cell-surface antigenic marker for oligodendrocytes in culture. *Nature*.

[61] Yuan X., Chittajallu R., Belachew S., Anderson S., McBain C. J., Gallo V. (2002). Expression of the green fluorescent protein in the oligodendrocyte lineage: a transgenic mouse for developmental and physiological studies. *Journal of Neuroscience Research*.

[62] Radtke C., Sasaki M., Lankford K. L., Gallo V., Kocsis J. D. (2011). CNPase expression in olfactory ensheathing cells. *Journal of Biomedicine and Biotechnology*.

[63] Gravel M., Peterson J., Yong V. W., Kottis V., Trapp B., Braun P. E. (1996). Overexpression of 2′,3′-cyclic nucleotide 3′-phosphodiesterase in transgenic mice alters oligodendrocyte development and produces aberrant myelination. *Molecular and Cellular Neurosciences*.

[64] Yin X., Peterson J., Gravel M., Braun P. E., Trapp B. D. (1997). CNP overexpression induces aberrant oligodendrocyte membranes and inhibits MBP accumulation and myelin compaction. *Journal of Neuroscience Research*.

[65] Lee S., Shea T. B. (2014). The high molecular weight neurofilament subunit plays an essential role in axonal outgrowth and stabilization. *Biology Open*.

[66] Chen X., Nelson C. D., Li X. (2011). PSD-95 is required to sustain the molecular organization of the postsynaptic density. *The Journal of Neuroscience*.

[67] Greene L. A., Tischler A. S. (1976). Establishment of a noradrenergic clonal line of rat adrenal pheochromocytoma cells which respond to nerve growth factor. *Proceedings of the National Academy of Sciences of the United States of America*.

[68] Bunge R. P., Bunge M. B., Bates M. (1989). Movements of the Schwann cell nucleus implicate progression of the inner (axon-related) Schwann cell process during myelination. *The Journal of Cell Biology*.

[69] Bauer G., Dao M. A., Case S. S. (2008). In vivo biosafety model to assess the risk of adverse events from retroviral and lentiviral vectors. *Molecular Therapy*.

[70] Martin G. R. (1981). Isolation of a pluripotent cell line from early mouse embryos cultured in medium conditioned by teratocarcinoma stem cells. *Proceedings of the National Academy of Sciences of the United States of America*.

[71] Shih C.-C., Forman S. J., Chu P., Slovak M. (2007). Human embryonic stem cells are prone to generate primitive, undifferentiated tumors in engrafted human fetal tissues in severe combined immunodeficient mice. *Stem Cells and Development*.

[72] Sieber-Blum M., Schnell L., Grim M., Hu Y. F., Schneider R., Schwab M. E. (2006). Characterization of epidermal neural crest stem cell (EPI-NCSC) grafts in the lesioned spinal cord. *Molecular and Cellular Neuroscience*.

